# Examining the relationship between therapeutic self-care and adverse events for home care clients in Ontario, Canada: a retrospective cohort study

**DOI:** 10.1186/s12913-017-2103-9

**Published:** 2017-03-14

**Authors:** Winnie Sun, Diane M. Doran, Walter P. Wodchis, Elizabeth Peter

**Affiliations:** 1 Faculty of Health Sciences, University of Ontario Institute of Technology, 2000 Simcoe St N, Oshawa, ON L1H 7K4 Canada; 2 Lawrence S. Bloomberg Faculty of Nursing, University of Toronto, 155 College Street, Suite 130, Toronto, ON M5T 1P8 Canada; 3 Institute of Health Policy Management and Evaluation, University Toronto, 155 College Street, 4th floor, Toronto, ON M5T 3M6 Canada

**Keywords:** Home care safety, Adverse events, Self-care, Self-management

## Abstract

**Background:**

In an era of a rapidly aging population who requires home care services, clients must possess or develop therapeutic self-care ability in order to manage their health conditions safely in their homes. Therapeutic self-care is the ability to take medications as prescribed and to recognize and manage symptoms that may be experienced, such as pain. The purpose of this research study was to investigate whether therapeutic self-care ability explained variation in the frequency and types of adverse events experienced by home care clients.

**Methods:**

A retrospective cohort design was used, utilizing secondary databases available for Ontario home care clients from the years 2010 to 2012. The data were derived from (1) Health Outcomes for Better Information and Care; (2) Resident Assessment Instrument-Home Care; (3) National Ambulatory Care Reporting System; and (4) Discharge Abstract Database. Descriptive analysis was used to identify the types and prevalence of adverse events experienced by home care clients. Logistic regression analysis was used to examine the association between therapeutic self-care ability and the occurrence of adverse events in home care.

**Results:**

The results indicated that low therapeutic self-care ability was associated with an increase in adverse events. In particular, logistic regression results indicated that low therapeutic self-care ability was associated with an increase in clients experiencing: (1) unplanned hospital visits; (2) a decline in activities of daily living; (3) falls; (4) unintended weight loss, and (5) non-compliance with medication.

**Conclusions:**

This study advances the understanding about the role of therapeutic self-care ability in supporting the safety of home care clients. High levels of therapeutic self-care ability can be a protective factor against the occurrence of adverse events among home care clients. A clear understanding of the nature of the relationship between therapeutic self-care ability and adverse events helps to pinpoint the areas of home care service delivery required to improve clients’ health and functioning. Such knowledge is vital for informing health care leaders about effective strategies that promote therapeutic self-care, as well as providing evidence for policy formulation in relation to risk mitigation in home care.

**Electronic supplementary material:**

The online version of this article (doi:10.1186/s12913-017-2103-9) contains supplementary material, which is available to authorized users.

## Background

In recent years, there has been an increasing amount of health resources devoted to home care settings for the management of disease conditions [1]. Home care is now one of the fastest growing programs in the Ontario health care system. Annually, home care services are received by more than 600,000 Ontario patients, 60% of which are seniors (ages 65 and over), including 27 million hours of personal support and homemaking, 6.5 million nursing visits and 1.9 million hours of nursing shifts [2]. Given the aging of the population, and the trend towards reduced use of institutionalized care settings, the number of clients being cared for at home will increase in the future. Therefore, it is vital to ensure that adequate home care resources are available to support those who wish to remain at home.

Home is an unpredictable site of care because home care providers are usually in the home for very short durations, and therefore home care requires the active involvement of the clients and their families [3]. Client outcomes are dependent on the quality of self-care provided by clients and their families, in addition to that provided by health care professionals [4]. Despite the growing reliance on home care, qualitative studies performed in the United States, Canada and Australia demonstrated that clients were unprepared for self-care in their homes after hospital discharge [5]. For instance, the clients reported that they had minimal input into their care plan, and they received conflicting advice regarding the self-care of their chronic illnesses. In addition, research on hospital readmissions found 9 to 48% of readmissions were associated with inadequate post-hospital discharge care [6]. A review of the current research evidence suggested that breakdowns in care from hospital to home could lead to increased utilization of health care resources, and negatively affect the quality of life and safety of clients and their informal caregivers [7].

As hospitals are discharging patients sooner and sicker, home care will play an important role in restoring and encouraging self-care in clients with chronic conditions. Self-care skills that are lost or not used as a result of health breakdown must be regained [8]. One of the best ways to reduce the impact of chronic conditions on people’s lives and on their need for expensive health care services is to support clients in the development of self-care skills. Providing self-care support to help clients manage their diseases is an important focus of home care services in the context of chronic disease management. However, the current trends in home care suggest that a greater proportion of home care resources have been directed towards post-hospitalized acute patients, with fewer resources available to support the long-stay clients with chronic health care needs [9]. As a result, many home care clients, especially older adults are at increased risk of losing independence in self-care, and this situation may put clients in unsafe situations, leading to safety problems or adverse events [10].

The current literature reveals gaps in care that raise concern about the safety of Canadian home care clients, and highlight the need for increased self-care support in home care. A scoping review of adverse events experienced by home care clients reported the overall rates of 3.5 to 15.1% which include the types of events such as adverse drug events, infections, wounds, and falls [11]. A recent Pan-Canadian Home Care Safety study concluded that 13% of clients in home care experience an adverse event annually [12]. Injurious falls were determined to be the most frequent adverse events and were associated with increased admission to long-term care or death. More specifically, Blais et al. determined that the decline in activities of daily living or instrumental activities of daily living were important indicators of frailty and were found to be associated with the increased odds of adverse events in home care [13]. The results revealed that more comorbid conditions (OR1.15; 95% CI 1.05 to 1.26) and a lower instrumental activities of daily living score (OR 1.54; 95% CI 1.16 to 2.04) were associated with having higher risk of experiencing an adverse event. As home care clients became more dependent and were more functionally vulnerable, they were at a greater risk of experiencing an adverse event. These findings point to the role of self-care ability as a potential contributing factor to adverse event. In particular, these findings raise the question to what extent the individual’s ability in therapeutic self-care is associated with the occurrence of adverse events in home care.

In an era of a rapidly increasing number of older people who require home care services, clients must possess or develop therapeutic self-care ability in order to manage safety in their homes. Therapeutic self-care is a concept developed by Sidani and Doran [14] to expand the understanding of self-care practice. It is defined as the ability to manage medications and treatment; to recognize signs and symptoms; to carry out treatments as prescribed; as well as having the knowledge of what to do in case of an emergency [15]. To date, little is known about the concept of therapeutic self-care in the context of home care safety. There is a lack of research that explores the relationship between the client’s self-care ability and safety problems in the home care settings. Our research addressed this existing gap by using an exploratory research approach to examine the role of therapeutic self-care ability in mitigating the risk of adverse events in home care.

The purpose of this research study was to examine the relationship between home care clients’ therapeutic self-care scores and adverse events. In particular, the study question was to evaluate the relationship between therapeutic self-care, adverse events, and health system utilization; while controlling for client demographics and clinical characteristics. This question provided insight into whether therapeutic self-care ability predicted variation in the frequency and types of adverse events experienced by the home care clients, such as client falls, emergency room visits and unplanned hospitalizations.

## Methods

### Conceptual framing: therapeutic self- care and adverse events

Self-care is viewed as encompassing a broad set of practices that individuals perform on their own behalf for the purposes of maintaining quality of life and well-being [16]. Therapeutic self-care is viewed as a sub-concept of self-care as it entails the level of knowledge and skill needed to support the self-care practice of disease management [17]. Sidani and Doran defined therapeutic self-care as the clients’ knowledge and ability to manage their health condition, manage symptoms, and follow the prescribed treatments [15]. Therapeutic self-care focuses on health deviation requisites in which changes in health condition demand actions to manage, control, and prevent them. Examples of managing health deviation requisites are self-monitoring and symptom management, including monitoring of specific physiologic parameters or symptoms of a health condition; adjustment to activities of daily living, seeking care as needed, and participating in treatment [16]. The domains of therapeutic self-care activities include the following areas: client’s knowledge of the prescribed medications and treatment; ability to recognize signs and symptoms; skills to carry out treatments as prescribed, and knowledge of what to do in case of an emergency [17].

The World Health Organization (WHO) [18] defined adverse events as the incidents that result in harm to a client. WHO [18] considers health care-associated harm as “harm associated with plans or actions taken during the provision of health care rather than an underlying disease or injury” (p. 8). The WHO definition of adverse events is useful for studying safety in the home care context because it recognizes that health care is not limited to medical care provided by health professionals, and it includes self-care [19]. In the literature adverse events are usually in reference to a breakdown in the process of care by the health care system. However, Lang et al. proposed that the safety of the home care client, informal caregiver, and health care providers are closely linked [20]. This recognition is consistent with the context of home care, where much of care is provided by clients and informal caregivers.

This study focused on clients and their informal caregivers as the care system in home care, and examined the occurrence of two types of outcomes that were most likely sensitive to therapeutic self-care ability: (1) use of health care resources, including new emergency room visits and unplanned hospital admissions; and (2) adverse events, including client falls; unintended weight loss; new urinary tract infection; activities of daily living (ADL) decline; non-compliance with medications; new pressure ulcer or ulcer deterioration; and new caregiver distress. These adverse events were examined for the following two reasons. First, these adverse events were expected to be sensitive to the clients’ therapeutic self-care ability as identified by Blais et al., Lang et al, and Sears et al. [13, 21, 22]. Second, these two types of outcomes are the most prevalent adverse events identified in the past home care safety research by Doran et al. [14, 21], including client falls (11%), emergency room visits (7%), new hospital visits (8%), unintended weight loss (9%), new urinary tract infection (8%), medication related incidents (2%) and new caregiver distress (11%). The operational definitions of adverse events are included in Additional file 1.

### Study design, setting and cohort

A retrospective cohort design was used to investigate the prevalence and types of adverse events that were associated with home care clients’ ability in therapeutic self-care in Ontario, Canada. In Ontario, clients generally receive home care services through the Community Care Access Centres (CCACs). The CCACs are single-point entry agencies that determine eligibility for the community and institutional services. They also act as case management organizations contracting with the home care agencies to provide services including professional nursing, therapies and homemaking. The cohort consisted of the populations of home care clients who received publicly funded home care services from the province between the periods of April 1, 2010 to March 31, 2012. The study population consisted of long-stay home care clients who were adults with the age of 18 years or older. Long-stay home care clients were defined as those who were expected to receive home care services for 60 days or longer.

### Ethics, data sources and linkages

The study received ethics approval from the University of Toronto Health Sciences Research Ethics Board. Ethics approval was obtained from the Institute of Clinical and Evaluative Sciences (ICES) where secondary data were derived from the following data sources: 1) HOBIC-HC (Health Outcomes for Better Information and Care-Home Care); 2) Home Care Reporting System RAI-HC (Resident Assessment Instrument-Home Care); 3) NACRS (National Ambulatory Care Reporting System) for emergency admissions; 4) DAD (Discharge Abstract Database) for hospital admissions. ICES staff ensured all data were made anonymous by removing personal identifiers prior to sharing the data with the researchers.

This study involved using HOBIC-HC assessments to investigate the independent effect of Therapeutic Self-Care (TSC) on the occurrence of adverse events. HOBIC assessments are collected to capture standardized client outcomes data related to nursing care in four sectors: acute care, long-term care, complex continuing care and home care [23]. The focus of this study was the HOBIC-HC Therapeutic Self-Care. Home care nurses are required to complete HOBIC documentation, including administering the Therapeutic Self-Care scale with their clients on admission and discharge.

The Therapeutic Self-Care scale is a 12-item instrument that measures the domains of self-care: taking prescribed medications; recognizing and managing symptoms; performing and adjusting regular activities; and managing changes in condition [14]. The items measure the clients’ perceived ability to perform these self-care activities with a 5-point numeric rating scale anchored with “not at all” through to “very much”, with higher total scores indicating high levels of self-care ability. The Therapeutic Self-Care scale has been used and applied in various clinical settings, particularly the scale showed reliability and validity in measuring self-care among individuals with traumatic injuries [24]. Further, there were three studies in the acute care setting where it demonstrated acceptable construct validity and internal consistency reliability based on Cronbach’s alpha of 0.88 [25–27]. The 12-item questions used in the Therapeutic Self-Care Scale are shown in Additional file 2.

RAI-HC assessments were used to provide information on client demographics and clinical characteristics, including age, sex, education; client living situation; comorbidities, the Cognitive Performance Scale; the Activities of Daily Living (ADL) Hierarchy score; the Depression rating score; pain scale and the Changes in Health, End-Stage Disease, and Symptoms and Signs Scale (CHESS). The assessments were also used to provide information on other adverse events, including (1) client falls; (2) unintended weight loss; (3) new urinary tract infection; (4) ADL decline; (5) new pressure ulcer or ulcer deterioration; (6) compliance/adherence to medications; and (7) new caregiver distress. The RAI-HC assessment provides a comprehensive profile of Canadian home care clients, their environment, services and outcomes [13]. The assessments are completed by the case manager at the Community Care Access Centre to evaluate the care needs of long-stay home care clients in Ontario. The psychometric properties of the RAI-HC can be found in Landy et al. and Morris et al. [28, 29].

The therapeutic self-care scores from HOBIC assessments were examined in association with the National Ambulatory Care Reporting System (NACRS) to investigate the relationship between therapeutic self-care and emergency room visits (ER visits) during the follow-up period. NARCS is a data system designed to capture information on client visits to facility and community-based ambulatory care in which data about visits are collected at the time of service in participating facilities [30]. This system is designed to provide valuable information that can help evaluate the management of ambulatory services in Canadian health care facilities. The therapeutic self-care scores from HOBIC assessments were examined in association with the Discharge Abstract Database (DAD) to investigate the relationship between therapeutic self-care and client health system utilization, specifically, acute care hospital admissions during the follow-up period. The DAD contains clinical and administrative data relating to health care services provided to the clients. The DAD is a record of hospital activity that is completed for each instance of a hospital separation, including discharge, death, and transfer to another facility [31].

The study cohort was constructed by linking HOBIC-HC assessments and baseline RAI –HC assessments using a common variable called Identification Key Number (IKN). The accrual of study cohort started from April 1, 2010 and ended on September 30, 2011, which resulted in a final sample of 1470 linked HOBIC-HC and baseline RAI-HC assessments. After the creation of study cohort, the 1470 linked assessments were followed-up for a 12-month period to investigate the occurrence of adverse events. The 1,470 HOBIC-HC assessments on therapeutic self-care measures were linked with the data from: (1) DAD; (2) NACRS; (3) subsequent RAI-HC assessments to determine adverse events. The baseline RAI-HC assessment was used to measure the client clinical characteristics and to look back in time for prior health system utilization. Subsequent RAI-HC assessments were used in the follow-up period, and were linked with the therapeutic self-care score to examine the occurrence of adverse events and their relationship with therapeutic self-care.

### Data analysis

Therapeutic self-care scores were the independent variables that were posited to influence adverse events in home care. The reliability of the multi-item Therapeutic Self-Care Scale was assessed using Cronbach’s alpha. A Cronbach’s alpha of 0.88 was found in an earlier study by Doran et al. in which the Therapeutic Self-Care Scale was correlated with ability to resume activities of daily living and role activities at hospital discharge, supporting its construct validity [27]. In this study, the Therapeutic Self-Care scale in the home care setting was tested for internal consistency reliability where the results indicated acceptable reliability based on Cronbach’s alpha of 0.97. Descriptive statistics were used to describe the client population characteristics such as gender, age, baseline and subsequent RAI-HC assessment data. Logistic regression analysis was used to determine the relationship between HOBIC therapeutic self-care scores and adverse events experienced by home care clients.

The frequency distribution of the 12-item therapeutic self-care scores indicated that the distribution of scores was skewed with high numbers of scores 5 (45%) for our study cohort. A decision was made to dichotomize this continuous variable into a binary variable by creating two groups for comparison in order to address the skewed distribution of therapeutic self-care scores. A final decision was made to use the cut-off value of score 5 by dichotomizing the therapeutic self-care score into a low self-care group (score 0 to 4) and a high self-care group (score 5). The cut-off value of score 5 is most appropriate to differentiate between high and low therapeutic self-care ability by creating a balance between the low self-care group (820 individuals) and high self-care group (650 individuals). Sensitivity analyses were performed with different cut-off values including the scores 5, 4 and 3. Results of sensitivity analyses are presented in Table 9. The findings of the analyses revealed stability of results across the different cut-off values.

The independent variables that were entered included the risk adjustment variables followed by the binary variables of low and high therapeutic self-care, as well as interaction terms. Risk adjustment was undertaken to adjust for different populations of clients who may be at greater risk of experiencing a poor outcome as a function of their demographic characteristics and clinical status [32]. A complete list of risk adjustment variables included in the models can be found in Additional file 3. The logistic regression models were developed using backward stepwise selection, which involved dropping statistically non-significant risk factors from the regression model. This method was selected because stepwise selection dealt directly with redundancy and helped identify those client risk factors that strongly affected the probability of the outcome [33]. While the non-significant risk factors (*p* > 0.1) were dropped from the model, age and gender were the two variables that were always retained in each model. The reason for keeping age and gender in the model was that past research has found these two variables were considered important when adjusting for biological differences among individuals [21, 32, 34, 35].

## Results

### Characteristics of study cohort

The results that describe the characteristics of study cohort can be found in Table 1. The 1470 study cohort consisted of a majority of home care clients who were female and were in the age range of 65+ with the average age of 71.9 years (standard deviation 14.9). During the 1-year follow-up period, 48.8% of home care clients had a new hospital visit that was urgent or a non-elective admission with an overnight stay, while 56.9% of home care clients had a new emergency room visit. There were 607 individuals out of 1470 study cohort who had a subsequent RAI-HC assessment that allowed for follow-up on the occurrence of adverse events. Individuals who did not have a subsequent RAI-HC assessment were excluded from the final data analysis. These individuals included 81 home care clients were admitted to long-term care facility and 230 individuals died during the 1-year follow-up period.

**Table 1:** Characteristics of study cohort and follow-up from 2011 to 2012

Cohort Characteristics	*N* = 1470	Percent
Age over 65	1025	69.8
Age over 75	734	49.9
Female	832	56.6
With Subsequent RAI-HC Assessments	615	41.8
Long-Term Care Admissions in 1 year follow-up	81	5.5
Death in 1 year follow-up	230	15.6
New Hospital Visits	717	48.8
New ER Visits	836	56.9

### Therapeutic self-care: high self-care vs. low self-care group

The study cohort consisted of 820 individuals who were in the low self-care group and 650 individuals who were in the high self-care group. The majority were female who were over the age of 65. Seventy-seven percentage of the study cohort were living with their informal caregivers at home. The low self-care group was found to be more functionally dependent in nearly all the measures. For example, the low self-care individuals were characterized as having more complexity in clinical status such as having recent hospitalizations; multiple chronic diseases, polypharmacy; physical symptoms such as edema; and higher CHESS (Changes in Health, End-Stage Disease, Signs and Symptoms) scores. The CHESS score is a composite measure of change in health status, end-stage disease and symptoms and signs (e.g. vomiting, dehydration, weight loss and shortness of breath), and has been shown to be a strong predictor of adverse events [36]. Further, the low self-care individuals demonstrated poor functional status with impaired self-reliance and difficulties with ADL activities such as mobility issues, as well as difficulties with Instrumental activities of daily living (IADL) such as medication management. They were found to be more cognitively impaired than the high self-care group, with depressive symptoms, and behavioral symptoms including the tendency to wander, be verbally abusive and be resistive to care.

### Prevalence of adverse events

New ER visits, new ADL decline, new hospital visits (any urgent/non-elective admission to hospital with an overnight stay), new client falls, and new caregiver distress were ranked among the top most frequently occurring adverse events. Unintended weight loss, non-compliance or adherence with medications, newly detected urinary tract infections, and new pressure ulcer/ulcer deterioration were less frequently identified events. The prevalence rates of adverse events identified in RAI-HC, DAD and NACRS for the home care clients from the year of 2011 to 2012 are presented in Table 2.

**Table 2:** Prevalence rates of adverse events identified in RAI-HC, DAD and NACRS for Home Care Clients from 2011 to 2012

Adverse event	N = Number of home care clients follow-up from 2010 to 2011	n = Number of home care clients with adverse events	Prevalence Rates % (n/N)
New ER Visit	1470	836	56.9%
New ADL Decline	615	318	51.7%
New Hospital Visit	1470	717	48.8%
New Fall	615	215	35.0%
New Caregiver Distress	615	166	27.0%
Unintended Weight Loss	615	83	13.5%
Non-Compliance/Adherence with Medications	615	68	11.0%
Newly Detected Urinary Tract Infection	615	40	6.5%
New Pressure Ulcer/Ulcer Deterioration	615	34	6.0%

### Association between therapeutic self-care and adverse events

Each logistic regression model was built using a backward stepwise selection process for the nine adverse events: (1) use of health care resources, including new emergency room visits and new hospital visits; (2) adverse events, including ADL decline; new falls; unintended weight loss; non-compliance/adherence with medication; new urinary tract infections; new pressure ulcers or ulcer deterioration; and new caregiver distress. The unadjusted and adjusted odds ratio estimates for therapeutic self-care in relation to adverse events are presented in Table 3.

**Table 3:** Unadjusted and adjusted odds ratio estimates for therapeutic self-care in relation to adverse events

Adverse events	Odds Ratio (OR) for therapeutic self-care	95% confidence interval	*p*-value
New Hospital Visit	Unadjusted OR 0.63	0.56, 0.96	0.03
	Adjusted OR 0.74		
New ADL Decline	Unadjusted OR 0.60	0.40, 0.84	0.04
	Adjusted OR 0.58		
New Fall	Unadjusted OR 0.63	0.42, 0.99	0.05
	Adjusted OR 0.64		
Unintended Weight Loss	Unadjusted OR 0.52	0.34, 0.99	0.05
	Adjusted OR 0.58		
Non-Compliance/Adherence with Medication	Unadjusted OR 0.46	0.25, 0.78	<0.01
	Adjusted OR 0.45		
New ER Visit	Unadjusted OR 1.25	0.97, 1.53	0.09
	Adjusted OR 1.22		
Newly Detected Urinary Tract Infection	Unadjusted OR 1.47	0.90, 3.36	0.09
	Adjusted OR 1.74		
New Pressure Ulcer/Ulcer Deterioration	Unadjusted OR 2.62	0.72, 13.87	0.13
	Adjusted OR 3.15		
New Caregiver Distress	Unadjusted OR 1.11	0.89, 2.00	0.16
	Adjusted OR 1.34		

The results of the logistic regression models revealed that home care clients’ use of health care resources was found to have association with therapeutic self-care scores. More specifically, the high self-care group was found to have a lower likelihood of new hospital visits than the low self-care group. The findings revealed that 47.4% of home care clients with high therapeutic self-care ability had unplanned hospital visits in comparison to 53.2% of individuals in the low self-care group. The result of the analyses indicated that the odds of having hospital visits for the high self-care group were 26% lower than the low self-care group. The results also indicated that the client factors that increased the odds of hospital visits were: being male, having an increase in heath instability as indicated by high CHESS scores, as well as having the diagnoses of Congestive Heart Failure or Alzheimer’s disease. In particular, polypharmacy (taking nine or more medications) and therapeutic self-care was found to be significant, which indicated that there was interaction between polypharmacy and therapeutic self-care ability in the occurrence of new hospital visits. High polypharmacy was associated with the increased odds of experiencing new hospital visits among the individuals with low therapeutic self-care ability. Sixty-one percent of home care clients with polypharmacy were found to have unplanned hospitalizations in this study cohort. The interaction model with adjusted odds ratio estimates for therapeutic self-care in relation to hospital visit is presented in Table 4.

**Table 4:** Interaction model with adjusted odds ratio estimates for therapeutic self-care in relation to hospital visit

Variables	Adjusted odds ratio	95% confidence interval	*p*-value
Therapeutic Self-Care	0.74	0.56, 0.96	0.03
Over age 75	1.01	0.77, 1.34	0.93
Female	0.77	0.63, 0.96	0.02
Polypharmacy	1.11	0.87, 1.44	0.40
Polypharmacy*Therapeutic Self-Care	1.48	1.10, 2.02	0.01
CHESS Scores	1.42	1.14, 1.76	<0.01
Congestive Heart Failure	1.66	1.20, 2.30	<0.01
Alzheimer’s Disease	1.31	1.01, 1.70	0.02
Model Fit Assessment	Chi-Square	DF	p-value
Likelihood Ratio	68.28	10	<0.01
Model Fit Assessment	Association of Predicted Probabilities and Observed Responses		
C-index	0.62		

New ADL decline, new client falls, unintended weight loss, and non-compliance with medication were the four adverse events that were found to have associations with therapeutic self-care scores. In particular, the low therapeutic self-care group was associated with an increased odds of ADL decline, new falls, unintended weight loss and non-compliance/adherence with medication. In summary, there were five adverse events that were found to have associations with therapeutic self-care scores: (1) new hospital visits; (2) new ADL decline; (3) new falls; (4) unintended weight loss; and (5) non-compliance with medication. In particular, the high therapeutic self-care group was associated with the decreased odds of adverse events, whereas the low therapeutic self-care group was associated with the increased occurrence of a hospital visit, ADL decline, falls, unintended weight loss and non-compliance with medication. The Tables 3, 4, 5, 6, 7 and 8 summarizes the logistic regression analyses with adjusted odds ratio estimates for therapeutic self-care scores in relation to the five adverse events, while the results of sensitivity analyses are presented in Table 9.

**Table 5:** Final logistic regression model with adjusted odds ratio estimates for therapeutic self-care in relation to new client fall

Variables	Adjusted odds ratio	95% confidence interval	*p*-value
Therapeutic Self-Care	0.64	0.42, 0.99	0.05
Over age 65	0.67	0.45, 1.00	0.05
Female	0.98	0.66, 1.45	0.92
Anti-depressant medications	1.63	1.07, 2.49	0.02
History of falls	1.95	1.16, 3.27	0.01
Model Fit Assessment	Chi-Square	DF	p-value
Likelihood Ratio	96.10	6	<0.01
Model Fit Assessment	Association of Predicted Probabilities and Observed Responses		
C-Index	0.72

**Table 6:** Final logistic regression model with adjusted odds ratio estimates for therapeutic self-care in relation to unintended weight loss

Variables	Adjusted odds ratio	95% confidence interval	*p*-value
Therapeutic Self-Care	0.58	0.34, 0.99	0.05
Over age 75	1.02	0.54, 1.95	0.95
Female	0.75	0.46, 1.24	0.27
Chess	2.15	1.26, 3.64	<0.01
Locomotion Outside of Home	2.58	1.39, 4.78	<0.01
Cancer	2.20	1.29, 3.75	<0.01
Model Fit Assessment	Chi-Square	DF	p-value
Likelihood Ratio	47.41	11	<0.01
Model Fit Assessment	Association of Predicted Probabilities and Observed Responses		
C-Index	0.73

**Table 7:** Final logistic regression model with adjusted odds ratio estimates for therapeutic self-care in relation to medication non-compliance

Variables	Adjusted odds ratio	95% confidence interval	*p*-value
Therapeutic Self-Care	0.46	0.26, 0.81	<0.01
Over age 75	1.75	1.14, 2.68	0.01
Female	1.52	1.07, 2.15	0.02
ADL Self-Performance	0.35	0.19, 0.65	<0.01
History of falls	1.82	1.01, 3.28	0.05
Difficulty in Managing Medications	2.53	1.35, 4.73	<0.01
Skin Problems	2.66	1.54, 4.59	<0.01
Model Fit Assessment	Chi-Square	DF	p-value
Likelihood Ratio	42.08	8	<0.01
Model Fit Assessment	Association of Predicted Probabilities and Observed Responses		
C-index	0.72

**Table 8:** Final logistic regression model with adjusted odds ratio estimates for therapeutic self-care in relation to new ADL decline

Variables	Adjusted odds ratio	95% confidence interval	*p*-value
Therapeutic Self-Care	0.58	0.40, 0.84	0.04
Over age 75	1.75	1.14, 2.68	0.01
Female	1.52	1.07, 2.15	0.02
Chess	2.21	1.55, 3.15	<0.01
History of falls	1.48	1.03, 2.14	0.03
Unsteady gait	1.82	1.21, 2.73	<0.01
Polypharmacy	1.23	1.13, 2.68	<0.01
Anxiolytic medications	1.56	1.02, 2.40	0.04
Model Fit Assessment	Chi-Square	DF	p-value
Likelihood Ratio	77.21	10	<0.01
Model Fit Assessment	Association of Predicted Probabilities and Observed Responses		
C-index	0.70

**Table 9:** Results of sensitivity analyses for adverse event: ADL decline

Variables	Adjusted odds ratio for therapeutic self-care	95% confidence interval	*p*-value
Therapeutic Self-Care (Using cut-off score 3)	0.58	0.39, 0.87	0.03
Therapeutic Self-Care (Using cut-off score 4)	0.64	0.44, 0.92	0.03
Therapeutic Self-Care (Using cut-off score 5)	0.58	0.40, 0.84	0.04

## Discussion

The prevalence rates of adverse events for home care clients were identified during the study period from 2011 to 2012. This research found the five most prevalent adverse events were: ADL decline (51.7%); client falls (35%); caregiver distress (27%); unintended weight loss (13.5%); and medication non-compliance (11%). Previous research found similar findings in which client falls, medication-related events and caregiver distress were the most frequent types of adverse events in home care observed through chart review and secondary data analysis during the year of 2008 and 2009 [13]. Our study findings provide a detailed description about the differences in the characteristics between low self-care and high self-care home care clients. This detailed description about the characteristics of clients with low therapeutic self-care ability is important to help identify the baseline characteristics of those individuals who are at greater risk for adverse events at home. There were five adverse events in home care that were found to be associated with therapeutic self-care ability: (1) New hospital visits; (2) ADL decline; (3) unintended weight loss; (4) client falls; and (5) medication non-compliance. Specifically, home care clients with low measured therapeutic self-care level were found to experience increased likelihood of having adverse events when compared with the individuals who possessed high therapeutic self-care ability.

The study findings suggest that the level of home care clients’ engagement in therapeutic self-care may be explained by the theory of patient activation. Patient Activation is the process that clients go through in becoming fully competent self-managers of their own health [37]. This process involves (1) believing that an active role is important; (2) having confidence and knowledge to take action; (3) taking action; and (4) staying the course under stress. Hibbard et al. proposed that being an engaged and active participant in one’s own care is associated with better health outcomes [38]. Clients who are engaged in self-care possess high level of self-sufficiency in caring for themselves, and thus they are better able to manage their health care needs [39]. Therefore, home care clients with a high level of therapeutic self-care ability were less likely to experience adverse events due to their active engagement in disease management. For example, home care clients with high levels of therapeutic self-care ability may have the necessary knowledge and skills to handle problems on their own at home, and access appropriate care to prevent unplanned hospitalizations. Individuals with high levels of therapeutic self-care ability may have the necessary knowledge to manage their health conditions and prevent further ADL decline.

These findings are consistent with Sidani and Doran’s definition of therapeutic self-care as the clients’ knowledge and ability to manage their health condition, manage symptoms, and follow the prescribed treatments [14]. The domains of therapeutic self-care activities include the following areas: clients’ knowledge of the prescribed medications and treatment; ability to recognize signs and symptoms; skills to carry out treatments as prescribed, and knowledge of what to do in case of an emergency [15]. Based on this conceptualization, therapeutic self-care is viewed as the knowledge and skill that facilitate the self-care practice related to health deviations. Therapeutic self-care ability enables clients to make informed choices regarding their self-care tasks and behaviors in relation to disease management [40]. This conceptualization suggests that individuals with low measured therapeutic self-care may demonstrate a lack of engagement in self-care and readiness for disease management. For example, home care clients with low therapeutic self-care ability may lack the knowledge and skills needed to maintain their health functioning to prevent unintended weight loss and risk for falls.

Similarly, individuals with low therapeutic self-care ability may lack the ability or knowledge to follow through on recommendations and comply with medication regimens. Therefore, the lack of self-sufficiency in therapeutic self-care may lead to the increased risk for adverse events among home care clients. This study’s conceptualization of therapeutic self-care recognizes that the notion of self-care is not only dependent upon the “self”, but it is also dependent on the support of other people [40]. For instance, our study data indicated that seventy-seven percent of the home care clients were living with their informal caregivers at home. This finding reveals the important role of informal caregivers in supporting the therapeutic self-care of home care clients. The safety of therapeutic self-care includes the responsibility of the individuals and their informal caregivers, and therefore those involved in the care of the home care clients need to be aware of the safety problems, and to develop the skills to mitigate those risks [41].

With regards to the use of health care resources, our study findings revealed that the prevalence rate for ER visits was 56.9% and the prevalence rate for hospital visits was 48.8%. These rates are consistent with the findings of recent home care studies in which researchers determined that unplanned visits to ER (60.5%) and unplanned admissions to hospital (38.3%) were among the most frequent adverse events for home care clients [13, 14]. Our study found that home care clients with high therapeutic self-care ability had lower odds of experiencing new hospital visits than clients with low therapeutic self-care ability. In particular, high polypharmacy was associated with the increased odds of experiencing new hospital visits among the individuals with low therapeutic self-care ability.

A possible explanation for these findings is that home care clients with a higher level of therapeutic self-care ability were more likely to become activated to engage in self-care practices. With a higher level of therapeutic self-care ability, clients may have the knowledge and skills needed to comply with medication regimens, and therefore may be more able to manage their polypharmacy. On the other hand, clients with a low therapeutic self-care level may lack the ability or knowledge to manage multiple medications, or access appropriate care to prevent health declines. Our findings are consistent with the polypharmacy literature that indicates the burden of taking multiple medications is associated with greater costs in health care utilization due to drug interactions, medication non-adherence and reduced functional capacity [41]. In the literature of Patient Activation, it was noted that individuals who were less engaged in self-care were found to be less compliant with drug regimens [36]. Lack of knowledge regarding one’s medication was found to be a contributing factor for the occurrence of medication non-compliance [42]. In particular, medication non-adherence in older adults has been associated with complicated medication regimens and polypharmacy [41]. The findings of this study point to the role of polypharmacy as one important contributing factor to the occurrence of unplanned hospitalizations, particularly among the individuals with low ability in medication management.

### Implications

Understanding home care clients’ risk profile is foundational to effective patient care management [19]. This study revealed the characteristics and risk factors associated with low therapeutic self-care individuals. This study also expanded the breadth of home care safety research by providing evidence regarding the types of adverse events that were associated with therapeutic self-care ability. For example, there was interaction between polypharmacy and therapeutic self-care in the occurrence of unplanned hospitalizations. Polypharmacy increased the incidence of potential drug-drug interaction which was found to be associated with medication-related hospitalizations in previous research [42, 43]. For example, drug interactions may cause a decline in functional ability in home care clients which compounded the risk of adverse events such as fall-related hospitalizations [14]. The Canadian Institute for Health Information found that those individuals with chronic conditions taking five or more prescription medications (13%) were more likely to experience a side effect requiring health care services than the individuals taking only one or two prescription medications (6%) [44]. Therefore, policies need to be developed to support best practices related to the management of polypharmacy at a health system level. For example, home care organizations need to develop guidelines and protocols for home care professionals in regards to medication reconciliation and frequency of medication review associated with polypharmacy. Reducing complex medication regimens to those necessary and aligned with client health goals should be central to medication management [45].

This study points out that the adverse events associated with therapeutic self-care ability could be potentially preventable, such as the occurrence of further ADL decline, client falls, unintended weight loss, and medication non-compliance. An understanding of the adverse events associated with therapeutic self-care ability should enable health leaders to make informed decisions about service priorities in home care settings. For example, the occurrence of unintended weight loss and medication non-compliance points to the need for service priorities to support clients’ IADL ability, including nutritional and medication management. Health care leaders need to allocate appropriate resources to support clients’ development of therapeutic self-care ability, as well as implementing appropriate measures in place that monitor clients’ risk for adverse events at home [40]. For example, home care organizations may adopt the use of HOBIC-HC therapeutic self-care scale as a tool to assess clients’ self-care ability and their risks for further ADL decline. Organizational policies need to be in place regarding modifications to home care environments to target the prevention of falls, as well as the implementation of best practice guidelines into clinical practice to assess the risk for falls and fall prevention strategies [46] to reduce injury and cost. Currently, the government of Ontario is implementing a new self-directed funding model for home and community care that is specific to the patients’ needs as they transition out of hospital and back in their homes [2]. It is imperative that these new quality improvement initiatives in home care services include inter-professional collaboration that facilitates patient safety through the enablement of therapeutic self-care in disease management.

## Conclusions

This is the first study that investigated the relationship between therapeutic self-care and adverse events in home care, and thus it makes an important contribution to the field. This research advances the understanding of the relationship between therapeutic self-care ability and the types and frequency of adverse events experienced by home care clients. High therapeutic self-care ability was found to be a protective factor against safety problems, while low self-care ability could be a risk factor in the occurrence of adverse events. This study highlights the importance of therapeutic self-care ability in influencing the occurrence of safety outcomes in the management of disease conditions at home. It also underscores the need for policy formulation that supports the development of therapeutic self-care ability in the prevention of adverse events for home care clients [40]. Our conceptualization of therapeutic self-care recognizes that the safety of self-care is not only dependent upon the individual, but it is also being supported by the informal caregivers. As a result, it is important that the supports and resources are provided to both the clients and their informal caregivers to improve the safety of home care.

This research study has a number of limitations. First, the study cohort of 1470 individuals is considered a small sample in secondary data research. The sample was small because there were a limited number of home care organizations submitting HOBIC-HC data to ICES at the time of the research. The sample size was further limited by including only long-stay clients who qualified for a RAI-HC assessment. Therefore, the study findings are only generalizable to the long-stay clients. Future research, involving more home care agencies across different geographical areas could further validate the evidence generated from the present study [40].

Another limitation of this research study is the under-reporting or over-reporting of the actual experience. For example, the frequency distribution of the HOBIC-HC therapeutic self-care scores was skewed with a high number of scores 5 for our study cohort. This situation may be due to the possibility that home care clients, informal or formal caregivers have over-reported or over-estimated the self-care ability of the individuals. Clients’ perceived ability to perform self-care behaviors is a subjective phenomenon [14]. Therefore, a response bias such as social desirability bias is a potential limitation when self-care is measured through the survey methods [47]. This situation was addressed in the analysis by dichotomizing the HOBIC therapeutic self-care scores into low self-care group (score 0 to 4) and high self-care group (score 5). Similar to other home care safety studies, there is no reliable way to determine with certainty whether the adverse events observed were due to the care delivered in the home or due to a client’s underlying diseases [14]. This limitation was addressed through the use of risk adjustment strategies to control for individual differences in the risk factors such as client characteristics or clinical status. Finally, there is the possibility that some adverse events were missed by the RAI-HC assessments because the periodical assessments are completed on average every 6 months. It is possible that not all adverse events could be detected at the time of follow-up RAI-HC assessments, and therefore our study results likely underreported the occurrence of adverse events [40]. Doran et al. highlighted some examples of adverse events in home care that are likely to be under-reported in the RAI-HC assessments, including non-recognition or non-reporting of medication-related problems; fall injuries that do not leave visible marks; or pressure ulcers and urinary tract infections that require clinical examination [13].

In summary, the findings revealed that a low level of therapeutic self-care ability could be a risk factor associated with the occurrence of adverse events in home care. The reasons why clients with high levels of therapeutic self-care were less likely to experience adverse events could be related to their active engagement in disease management. Therapeutic self-care ability is the level of knowledge and skill that enables clients to make informed choices regarding the management of their disease conditions. As a result, high therapeutic self-care ability is viewed as an enabling factor that could protect the clients against safety problems in the home care settings. It was important to study the enablement perspective of therapeutic self-care because of its potential role in risk mitigation, as well as its role in reducing the care burden of informal caregivers. The findings underscore the importance of assessing clients’ readiness for therapeutic self-care, and supporting their level of self-sufficiency in caring for themselves and managing their self-care needs at home. A clear understanding of the nature of relationship between therapeutic self-care ability and adverse events helps to pinpoint the areas of health care service delivery required to improve clients’ health and functioning [40]. Such knowledge is vital for informing health care leaders about the effective strategies that promote therapeutic self-care, as well as providing evidence for policy formulation in relation to risk mitigation that supports older adults and enables them to remain at home as long as possible.

## Additional files

Additional file 1: Operational definitions and data sources of adverse events. (DOC 33 kb)
Additional file 2: Therapeutic self-care scale - Home Care. (DOC 22 kb)
Additional file 3: Risk adjustment variables from RAI-HC. (DOC 57 kb)

## Abbreviations

ADL: Activities of daily living
AE: Adverse event
CCAC: Community Care Access Centre
CHESS: Changes in health, end-stage disease, signs and symptoms
DAD: Discharge abstract database
ER: Emergency room
HOBIC-HC: Health Outcomes for Better Information and Care-Home Care
IADL: Instrumental activities of daily living
ICES: Institute for Clinical and Evaluative Sciences
IKN: Identification key number
NACRS: National Ambulatory Care Repository System
RAI-HC: Resident Assessment Instrument-Home Care
TSC: Therapeutic self-care
WHO: World Health Organization
