# Supplementary material for: Examining the relationship between therapeutic self-care and adverse events for home care clients in Ontario, Canada: a retrospective cohort study
Source: BMC Health Serv Res. 2017 Mar 14;17:206. doi: 10.1186/s12913-017-2103-9 (PMC5351056; doi:10.1186/s12913-017-2103-9)
Supplement: Additional file 1: — Operational definitions and data sources of adverse events. (DOC 33 kb) [file 12913_2017_2103_MOESM1_ESM.doc]

**Additional file 1: Operational Definitions of Adverse Events**

| **Adverse Events** | **Operational Definitions** | **Coding** | **Sources of Data** |
| --- | --- | --- | --- |
| New hospital visit | Any admission to hospital with an overnight stay within one year following HOBIC assessment | 0 for none  1 for any | DAD |
| New emergency room visit | Any emergency room visit without an overnight stay within one year following HOBIC assessment | 0 for none  1 for any | NACRS |
| Client Fall | Number of times fell in last 90 days or since last assessment if less than 90 days | If none, code “0”, if more than 9, code “9” | RAI-HC  Variable name:  K5 |
| Unintended weight loss | Unintended weight loss of 5% or more in the last 30 days (or 10% or more in the last 180 days) | 0 for No  1 for Yes | RAI-HC  Variable name:  L1a |
| Newly detected urinary tract infection | Urinary tract infection in last 30 days | 0: not present  1 or 2: present | RAI-HC  Variable name: J1W |
| New caregiver distress | A caregiver is unable to continue in caring activities (e.g. decline in the health of the caregiver makes it difficult to continue); primary caregiver expresses feelings of distress, anger or depression | 0 for No  1 for Yes | RAI-HC  Variable name:  G2a and G2c |
| ADL decline | ADL status has become worse (ie. now more impairment in self-performance) as compared to status 90 days ago (or since last assessment if less than 90 days) | 0 for No  1 for Yes | RAI-HC  Variable name:  H3 |

| **Adverse Events** | **Operational Definitions** | **Coding** | **Sources of Data** |
| --- | --- | --- | --- |
| New pressure ulcer or Ulcer deterioration | Pressure ulcer appeared or stage increased at 2^nd^ assessment compared with previous assessment | 0 for No  1 for Yes | RAI-HC  Variable name:  N2a |
| Non- Compliance/  Adherence with Medications | Compliant all or most of time with medication prescribed by physician in last 7 days | 0 for Always compliant or compliant 80% of time or more  1 for compliant less than 80 % of time, including failure to purchase prescribed medications | RAI-HC  Variable name:  Q4 |
