# Supplementary material for: Examining the relationship between therapeutic self-care and adverse events for home care clients in Ontario, Canada: a retrospective cohort study
Source: BMC Health Serv Res. 2017 Mar 14;17:206. doi: 10.1186/s12913-017-2103-9 (PMC5351056; doi:10.1186/s12913-017-2103-9)
Supplement: Additional file 2: — Therapeutic self-care scale - Home Care. (DOC 22 kb) [file 12913_2017_2103_MOESM2_ESM.doc]

**Additional file 2**

**Home Care: THERAPEUTIC SELF-CARE SCALE (Sidani & Doran)**

12-item questions:

1. Do you know what medications you have to take?

2. Do you understand the purpose of the medications prescribed to you (that is, do you know what the medications do for your health condition)?

3. Do you take the medications as prescribed?

4. Can you recognize changes in your body (symptoms) that are related to your illness or health condition?

5. Do you know and understand why you experience some changes in your body (symptoms) related to your illness or health condition?

6. Do you know what to do (things or activities) to control these changes in your body (symptoms)?

7. Do you carry out the treatments or activities that you have been taught to manage these changes in your body (symptoms)?

8. Do you do things or activities to look after yourself and to maintain your health in general?

9. Do you know whom to contact to get help in carrying out your daily activities?

10. Do you know whom to contact in case of a medical emergency?

11. Do you perform your regular activities (such as bathing, shopping, preparing meals, visiting with friends)?

12. Do you adjust your regular activities when you experience body changes (symptoms) related to your illness or health condition?
