# Supplementary material for: Examining the relationship between therapeutic self-care and adverse events for home care clients in Ontario, Canada: a retrospective cohort study
Source: BMC Health Serv Res. 2017 Mar 14;17:206. doi: 10.1186/s12913-017-2103-9 (PMC5351056; doi:10.1186/s12913-017-2103-9)
Supplement: Additional file 3: — Risk adjustment variables from RAI-HC. (DOC 57 kb) [file 12913_2017_2103_MOESM3_ESM.doc]

**Additional file 3: Risk Adjustment Variables from RAI-HC**

| **Variable Name** | **Variable Code** | **Operational Definition** |
| --- | --- | --- |
| Edema | K3a | Problem condition |
| Diabetes | J1y | Disease diagnosis |
| Polypharmacy | Q1 | Taking more than 9 medications |
| Depression Rating Scale | DRS | Scale to measure depressive symptoms |
| Changes in Health, End-Stage Disease, Signs and Symptoms Scale | Chess | Composite measure of change in health status, end-stage disease and symptoms and signs (e.g. vomiting, dehydration, weight loss and shortness of breath) |
| Anti-depressant | Q2c | Receipt of psychotropic medication |
| Cognitive Performance Scale | CPS | Scale to measure level of cognitive impairment |
| Activity of Daily Living Self-Performance | ADL | Scale to address client’s physical functioning in routine personal activities of daily life, for example, dressing, eating etc. |
| Education | BB6 | Education(Highest level completed) |
| Instrumental Activity of Daily Living Self-Performance | IADL | Scale to measure level of difficulty when performing IADL tasks: meal prep, telephone use, ordinary housework, managing finances, managing medications, shopping and transportation |
| Transfer | H2b | Including moving to and between surfaces, to/from bed, chair, wheelchair, standing position |
| Wandering | E3a | Moved with no rational purpose, seemingly oblivious to needs or safety |
| Arthritis | J1m | Disease diagnosis: Musculoskeletal |
| Hip fracture | J1n | Disease diagnosis: Musculoskeletal |
| Cognitive skills for daily decision making | B2a | Cognitive Patterns: how well client made decisions about organizing the day |
| Other fractures | J1o | Other fractures (e.g. wrist, vertebral) |
| ADL decline | H3 | ADL status has become worse (i.e. now more impaired in self-performance) |
| Osteoporosis | J1p | Disease diagnosis: Musculoskeletal |
| Locomotion in home | H2c | Physical functioning in home |
| Locomotion outside of home | H2d | Physical functioning outside of home |
| Cancer | J1x | Disease diagnosis |
| History of falls frequency | K5 | History of number of times fell |
| Managing Medications | H1d | How medications are managed (e.g. remembering to take medicines, opening bottles, taking correct drug dosages, giving injections, applying ointments |
| Self-Reliance Index | SRI | The SRI categorizes clients as being either self-reliant or impaired. Self-reliance is based on being independent or requiring set-up help only on bathing, personal hygiene and walking, and being independent in cognitive skills for daily decision making. |
| Anxiolytic | Q2b | Receipt of psychotropic medication |
| Unsteady gait | K6a | Danger of falling |
| Informal helper lives with client | G1e | Informal support services |
| Verbally abusive behavioral symptoms | E3b | Threatened, screamed at, cursed at others |
| Physically abusive behavioral symptoms | E3c | Hit, shoved, scratched, sexually abused others |
| Socially inappropriate or Disruptive behavioral symptoms | E3d | Disruptive sounds, noisiness, screaming, self-abusive acts, sexual behavior or disrobing in public, smears/throws food/feces, rummaging, repetitive behavior, rises early and causes disruption |
| Resists care | E3e | Resisted taking medications/injections, ADL assistance, eating, or changes in position |
| Morbid obesity | L1c | Nutrition/hydration status: weight |
| Severe malnutrition | L1b | Nutrition/hydration status: weight |
| Skin problems | N1 | Any troubling conditions or changes in skin condition (e.g. burns, bruises, rashes, itchiness, body lice, scabies) |
| History of pressure ulcers | N2a | Any lesion caused by pressure, shear forces, resulting in damage of underlying tissues |
| Stroke | J1a | Disease diagnosis: heart/circulation |
| Congestive heart failure | J1b | Disease diagnosis: heart/circulation |
| Coronary artery disease | J1c | Disease diagnosis: heart/circulation |
| Hypertension | J1d | Disease diagnosis: heart/circulation |
| Alzheimer’s | J1g | Disease diagnosis: Neurological |
| Dementia other than Alzheimer’s disease | J1h | Disease diagnosis: Neurological |
| Any psychiatric diagnosis | J1s | Disease Diagnosis: Psychiatric/Mood |
| Parkinsonism | J1l | Disease Diagnosis: Neurological |
| Emphysema/COPD/Asthma | J1z | Disease Diagnoses |
